# Cyanoacrylate-induced pseudo-tarsorrhaphy of the eye

**DOI:** 10.4103/0974-620X.48421

**Published:** 2009

**Authors:** Upender Wali, Rana Al-Senawi, Abdullah Al-Mujaini

**Affiliations:** Department of Ophthalmology, Sultan Qaboos University Hospital, Muscat, Sultanate of Oman

**Keywords:** Abrasion, cyanoacrylate, pseudo-tarsor-rhaphy

## Abstract

Cyanoacrylate, also known as superglue, is a polymer which forms a strong bond at room temperature with a variety of materials including metal, glass, plastic, rubber, skin, mucous membranes and other epithelial tissues as well. We hereby present a 22-year-old female who had an accidental instillation of the glue in her eye, with an uneventful outcome.

## Introduction

Cyanoacrylate is a tenacious adhesive and has been explored for a wide variety of uses ranging from suture-less surgery to rock climbing to domestic and industrial use. Cyanoacrylate is a polymer which forms a strong bond at room temperature with a variety of materials including metal, glass, plastic, rubber, skin, mucous membranes and other epithelial tissues as well.[1]

## Case Report

**Figure 1:**
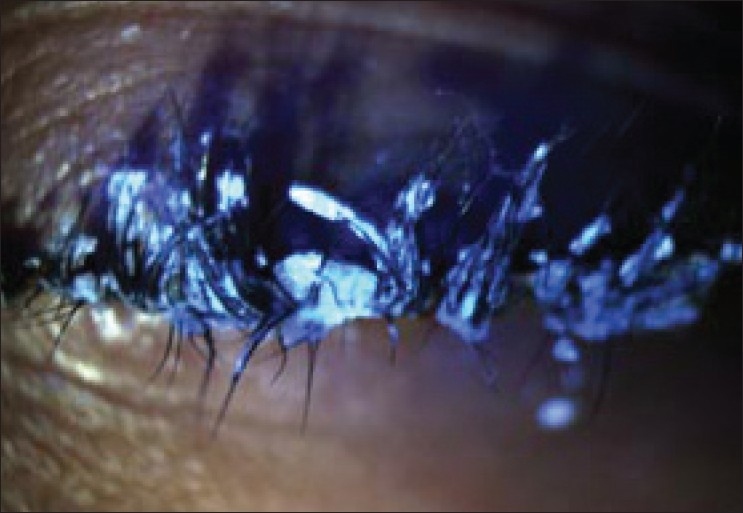
Cyanoacrylate (solidified superglue) on eyelashes: the lids could not be opened, nor could the glue be removed with forceps, giving the initial impression of a tarsorrhaphy

**Figure 2:**
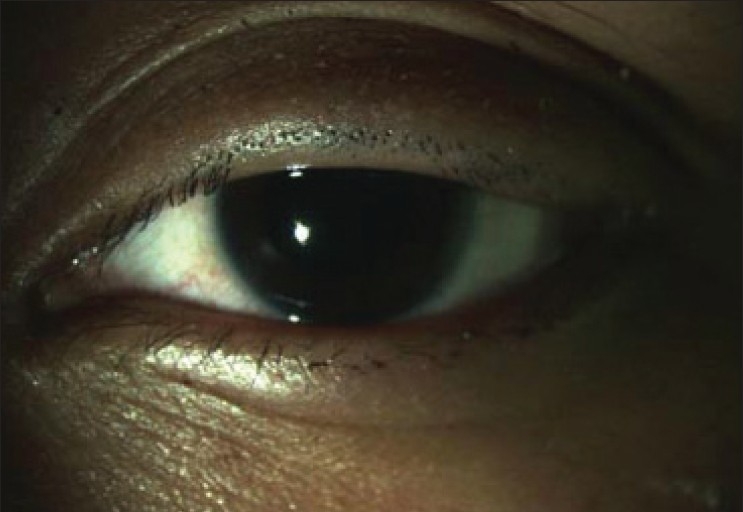
No skin or mucous membrane adhesions were found between the lids after trimming the eyelashes, proving that tarsorrhaphy was false (pseudo-tarsorrhaphy)

A 22-year-old female had cyanoacrylate accidentally instilled in her left eye. She had not pierced the top protection seal with the pin provided in the pack, instead kept on squeezing the tube till it burst open with pressure. She reported 24 hours after the incident. During this period no treatment was sought. On examination the eyelashes were glued together throughout their length with no gross visibility of the lid margins or the ocular structures [Figure 1]. Attempted removal of cyanoacrylate with forceps was painful. The idea of conservative observation was abandoned on patient‘s request as she had to take her examination three days later. It was decided to trim the entire rows of eyelashes with a scissor on slit-lamp. This resulted in spontaneous opening of the lids with no adhesions of the lid margin or mucous membrane [Figure 2]. Her cornea revealed a small abrasion inferiorly, which healed with an antibiotic ointment and a patch. Examination of the fornices did not reveal any residual glue. Subsequent follow up showed that the patient had no visual or morphological sequelae.

## Discussion

Eye injuries with cyanoacrylate have been reported where patient or attendant either mistook the substance tube with an ophthalmic medication[2] or a cosmetic item.[3] Trauma occurs mainly due to poor vision, carelessness, handling by children or poor room light. Our patient knew the nature and component of the tube, and its use, but surprisingly did not know that it had to be pierced with a pin from the top. Literature review did not reveal such a reason for accidental instillation of this material in the eye.

The management of cyanoacrylate eye injury is varied ranging from “leaving the eye to open spontaneously, to irrigation, saline padding, pressure patching, use of mineral oil, acetone swabs,[4] ointment, sodium bicarbonate, bandage contact lens, removal of the glue pieces with forceps, trimming of eye lashes and surgical separation of lids. The treatment modality is largely based on individual case, and remains overall same for all variants of cyanoacrylate (domestic, industrial and medical). One should always look for any residual glue in the fornices as it can cause infectious keratitis, giant papillary conjunctivitis, granulomatous conjunctivo-keratitis or punctuate epithelial erosions.[5] Earlier the lids are opened, better it is, as this enables the earlier examination of cornea and fornices, besides preventing any amblyopia in children.[5]

Some variants of cyanoacrylate result in a powerful and rapid exothermic reaction with cotton or wool (such as cotton balls, fabrics), sometimes causing fire. Normally it takes less than 2 minutes for the glue to become solid; during this period, no attempt should be made to remove the liquid glue with such material.[6] Once solidified, no such reaction occurs.

In summary, whatever the make of cyanoacrylate or the mode of its injury may be, the glue does not seem to cause any significant mechanical, chemical, thermal or toxic injury to the ocular structures. In spite of instructions provided in the cyanoacrylate pack, similarities in the packaging of cyanoacrylate nail glue and cosmetic/ophthalmic preparations is an ongoing problem.[7] Ways and methods should be innovated by the manufacturers to change the packing and make the use of cyanoacrylate as safe as possible.
